# Aging in the *Drosophila* ovary: contrasting changes in the expression of the piRNA machinery and mitochondria but no global release of transposable elements

**DOI:** 10.1186/s12864-019-5668-3

**Published:** 2019-04-23

**Authors:** Alexandra A. Erwin, Justin P. Blumenstiel

**Affiliations:** Department of Ecology and Evolutionary Biology, University of Kansas, Lawrence, KS 66045 USA

**Keywords:** Aging, Reproductive aging, Heterochromatin, Transposable element, Mitochondria, piRNA, *Drosophila melanogaster*, Ovary, Egg chamber

## Abstract

**Background:**

Evolutionary theory indicates that the dynamics of aging in the soma and reproductive tissues may be distinct. This difference arises from the fact that only the germline lineage establishes future generations. In the soma, changes in the landscape of heterochromatin have been proposed to have an important role in aging. This is because redistribution of heterochromatin during aging has been linked to the derepression of transposable elements and an overall loss of somatic gene regulation. A role for changes in the chromatin landscape in the aging of reproductive tissues is less well established. Whether or not epigenetic factors, such as heterochromatin marks, are perturbed in aging reproductive tissues is of interest because, in special cases, epigenetic variation may be heritable. Using mRNA sequencing data from late-stage egg chambers in *Drosophila melanogaster*, we characterized the landscape of altered gene and transposable element expression in aged reproductive tissues. This allowed us to test the hypothesis that reproductive tissues may differ from somatic tissues in their response to aging.

**Results:**

We show that age-related expression changes in late-stage egg chambers tend to occur in genes residing in heterochromatin, particularly on the largely heterochromatic 4th chromosome. However, these expression differences are seen as both decreases and increases during aging, inconsistent with a general loss of heterochromatic silencing. We also identify an increase in expression of the piRNA machinery, suggesting an age-related increased investment in the maintenance of genome stability. We further identify a strong age-related reduction in the expression of mitochondrial transcripts. However, we find no evidence for global TE derepression in reproductive tissues. Rather, the observed effects of aging on TEs are primarily strain and family specific.

**Conclusions:**

These results identify unique responses in somatic versus reproductive tissue with regards to aging. As in somatic tissues, female reproductive tissues show reduced expression of mitochondrial genes. In contrast, the piRNA machinery shows increased expression during aging. Overall, these results also indicate that global loss of TE control observed in other studies may be unique to the soma and sensitive to genetic background and TE family.

**Electronic supplementary material:**

The online version of this article (10.1186/s12864-019-5668-3) contains supplementary material, which is available to authorized users.

## Background

The age-related decline of the reproductive system has important consequences for evolution because reproductive success determines the fitness of an organism. Since the majority of aging studies focus on overall somatic decline, relatively less is known about the causes of reproductive aging. In humans, progressive delays in childbearing are leading more people to confront the reduced fertility and fecundity that accompanies advanced age [1, 2]. Reproductive senescence is not unique to mammals, however. The invertebrate model *Drosophila melanogaster* shows a progressive decline in egg production at middle age, thought to be partially caused by a reduction in germline stem cell proliferation and decreased survival of developing eggs [3]. Possible mechanisms underlying these changes include reduced ovariole number, decreased rates of germline stem cell division, and apoptosis in egg chambers of older females [3, 4]. Animals may have conserved mechanisms to regulate reproductive decline and control the relationship between reproduction and lifespan. Not only have mechanisms of gametogenesis been found to be similar across organisms, but the control of ovulation has also been shown to be conserved between *Drosophila* and humans [5]. Because *Drosophila* is an established model for studies of both reproductive and somatic aging, we used it here to examine age-related genome-wide expression changes in the female reproductive tissues.

While genetic causes have long been shown to determine longevity - through either inherited or somatic mutation, non-genetic contributions are also proving to be major factors. Epigenetic chromatin marks play an essential role in the maintenance of genome integrity through their repression of genes, repeat sequences, and transposable elements (TEs) (reviewed by [6]). The mis-regulation of epigenetic marks has been associated with many diseases, including kidney disease, neurodegenerative diseases, and cancer [7–9]. Recently, epigenetic mis-regulation has been attributed to playing a key role in the aging process. In particular, the landscape of silent heterochromatin has been shown to redistribute in aged stem cells and cells of the soma, leading to aberrant gene expression [10–15]. An additional consequence of this redistribution of heterochromatin is the observed derepression of TEs in the soma during aging, notably in brains and fat body of *Drosophila*, and in a variety of other organisms including mammals [16–20]. A recent study indicates that the aging related release of TE control may promote inflammation [21]. Strikingly, this pattern of increased TE expression in the aging soma can also vary in accordance with reproductive strategy. In termites, age-related increases in TE expression are evident in members of the worker caste. In old termite workers, increased TE expression is also associated with reduced expression of the piRNA machinery [22].

Although interesting for the biology of aging, somatic cells are not the source of future generations. In contrast, the germline is an immortal cell lineage and germ cells employ unique strategies to faithfully transmit DNA indefinitely. These include mechanisms such as greater telomerase maintenance [23] and greater resistance to genotoxic stress than somatic cells [24]. However, age-related changes in the germline certainly occur. For example, some germ cells lose the ability to divide and differentiate normally [3], the sperm of older human males are at increased risk for de novo mutations [25], and double-strand break repair in oocytes in humans and mice declines with age [26]. Additionally, age-dependent meiotic nondisjunction may be due to a loss of the cohesin complex that regulates the separation of sister chromatids over time [27–29]. However, little is known about the extent to which such aging-related changes in reproductive tissues are heritable and manifest across generations.

In the germline, aging can be partially attributed to intrinsic factors residing within the germline stem cell lineage. In addition, some germline age-effects can also be attributed to extrinsic factors such as the microenvironment of the germline stem cells [3, 4, 30]. The relative roles of extrinsic versus intrinsic factors in contributing to germline aging are still being explored. In mammals, much of the current evidence points to a greater role of cell-extrinsic factors. Similar to flies, niche deterioration also may play a role in the mammalian system [31]. For example, it has been shown that mammalian spermatogonial stem cells, when transplanted to a young environment, have extended functionality [32, 33]. Signaling factors such as insulin may also play a role in maintaining germline function in mammals [34, 35]. Thus, while the germline is generally considered to be immortal, components of the germline and its microenvironment are not resistant to age-related changes.

Recent findings highlighting the large role of chromatin-based changes in the somatic aging process leads us to question whether similar mechanisms may also be at play in reproductive tissues. Although the majority of epigenetic marks are erased and re-established between generations, some epigenetic modifications are transmitted across generations through the germline. Longevity itself is a trait that can be epigenetically inherited in *C. elegans* [36–38]. Of most relevance, *Drosophila* oocytes transmit the repressive histone mark H3K27me3 to their offspring [39]. This creates a potential for age-effects to be passed on to the next generation, an outcome that could pose new questions for traditional evolutionary theories of aging.

Few studies have characterized genome-wide, age-related expression in ovaries and we are not aware of any such studies in *Drosophila*. Using mRNA expression as a proxy, we sought to determine whether age-related changes in TE control and chromatin-based gene regulation occur in the ovary. We tested this hypothesis by determining whether expression of the piRNA machinery is diminished and whether TEs become derepressed during aging. We further tested the heterochromatin aging hypothesis by testing whether genes in or near heterochromatin boundaries become derepressed in aging reproductive tissues. Strikingly, we find that expression of piRNA machinery is increased in aged ovaries. This may explain the observation that aging can enhance TE silencing and suggests that investment in germline genome stability may increase as flies age. We also find that while gene expression changes are enriched in heterochromatic regions of the genome, the direction of change is not consistent with a global increase in expression of heterochromatin. Further, we only find idiosyncratic aging effects on TE expression and no global increase. Nonetheless, we find a consistent pattern of decreasing expression of mitochondrial transcripts, as has been observed in non-reproductive tissues [40]. These results suggest that the age-related transposon release and the heterochromatin aging hypothesis do not extend to the *Drosophila* ovary in a simple manner.

## Results

### A shared profile of ovarian aging across strains

A number of studies have compared the transcriptome during aging across tissues and even across species [41–46]. Fewer studies, however, compare profiles in more than one natural strain [47, 48]. Here we sought to determine how gene expression is modulated in the aging ovary in two different inbred Raleigh strains of *Drosophila melanogaster* obtained from the DGRP [49]. Since ovaries are highly heterogeneous, consisting of a mixture of somatic tissues, germline-stem cells and many different stages of oogenesis, we focused our RNA-seq analysis using stage 14 egg chambers. This allowed us to minimize variation of cell type composition and to enrich for age-effects in the germline. Stage 14 egg chambers consist of an oocyte surrounded by a follicular sheath and represent the last stage of oogenesis before fertilization and oviposition. To measure differences in gene expression, we compared expression profiles in stage 14 egg chambers from mothers at 3–4 and 32–34 days post-eclosion (sample overview presented in Table 1). Overall, we identified 300 transcripts that were differentially expressed (DE) between young and old stage-14 egg chamber samples in a combined analysis with the two Raleigh lines (FDR adjusted *p*-value <.05), testing for age while controlling for strain in DESeq2 (Additional file 3: Table S1).

**Table 1:** Sample overview of stage 14 egg chambers

DGRP Strains	# Biological Replicates (3-4d)	# Biological Replicates (32-34D)
Ral_321	4	4
Ral_237	3	3

Ral 237 and Ral 321 were the two DGRP strains utilized for RNA sequencing analysis. D = days post-eclosion. Each biological replicate is a pool of egg chambers from five females

**Fig. 1:**
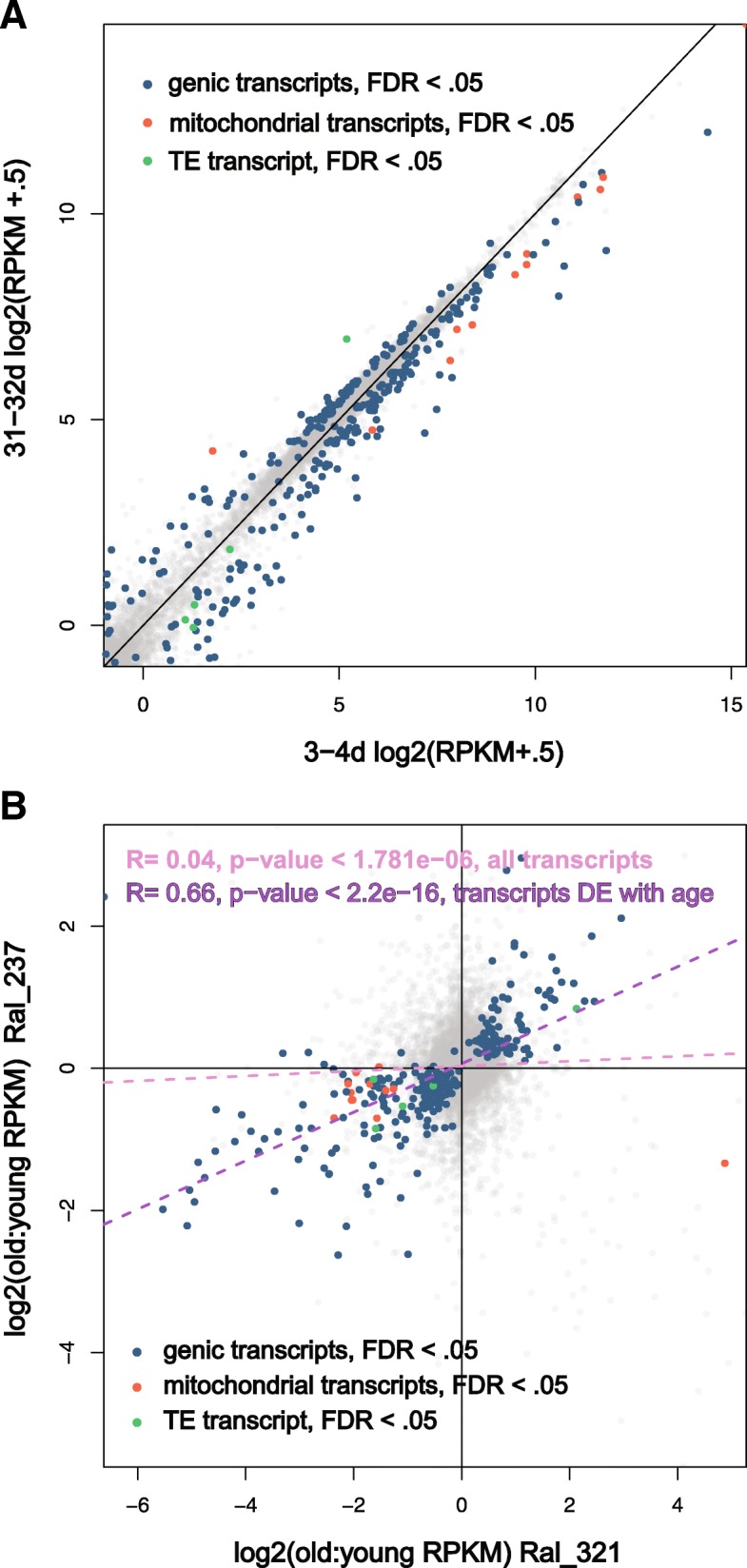
Signature of age-related expression in egg chambers across genetic background. (**a**) Average (between strains) log 2(RPKM+.5) expression of stage 14 egg chamber transcripts of old 30–34 day old samples versus young 3–4 day old samples. Transcripts significantly differentially expressed between young and old in a paired analysis (FDR < .05) are colored according to transcript type. Five TE transcripts are significantly differentially expressed across both strains with age, with only one, *copia*, showing an increase in expression. (**b**) Log2 ratios of old to young (RPKM+.5) expression between strains. The differentially expressed transcripts (FDR *p* < .05) are, as expected, strongly and significantly correlated with age across strains

Of the DE transcripts identified in the combined analysis, 106 transcripts show an average increase with age, while 194 show an average decrease with age across strains (Fig. 1a). Figure 1b demonstrates that the significantly differentially expressed transcripts, as fully expected, are strongly correlated between strains and show the same direction of expression changes between old and young egg chambers across the two strains (Pearson’s product-moment correlation = 0.66, *p*-value <1E-10). Seven of these genes have previously been associated with regulation of lifespan. Notably, *hebe* (CG1623) overexpression increases both longevity and fecundity [50] and *Hsp27* overexpression increases lifespan [51]. Both of these transcripts showed average lower expression in older stage-14 egg chambers across the two strains (*hebe*: Average 4.11-fold decrease, FDR *p*-value < 1.28E-05; *Hsp27*: Average 1.6-fold decrease; FDR p-value < 0.006). *Hsp27* was also one of the most highly expressed genes (26th). Another gene, *POSH* (Plenty of SH3s, CG4909) has been shown to promote cell survival in both *Drosophila* and human cells when overexpressed [52]. We find that this transcript shows a 1.46-fold increase with age in egg chambers (FDR adjusted *p*-value< 4.05E-05). The other DE transcripts previously associated with regulation of lifespan include Thiolase (CG4581), Thor (CG8846), Coq2 (Coenzyme Q biosynthesis protein 2; CG9613), and Tpi (triose phosphate isomerase; CG2171). Other notable categories of gene ontology were identified using GOrilla [53]. Results for biological process by rank significance include terms pertaining to the electron transport chain (GO:0022900; GO:0022904), mitochondrial electron transport chain (GO:0006120), numerous metabolic processes, developmental and cellular processes involved in reproduction (GO:0003006; GO:0022412), eggshell chorion assembly (GO:0007306) and many terms related to regulation of mitochondrial organization and fusion. Interestingly, even though we were examining reproductive tissues, the GO term for determinant of adult life span (GO:0008340) was also enriched. Full results from a gene ontology (GO) analysis for biological process, component, and function by rank significance is shown in Additional file 4: Table S2.

While these DE transcripts may provide a signature of senescence for egg chambers, the transcriptome, as a whole, shows only a very weak correlation in age-related patterns of expression across these two strains (Pearson’s product-moment correlation = 0.04, *p*-value < 1.8E-06, Fig. 1b). This demonstrates that many observed changes in gene expression in the aging ovary are likely to be strain specific. In fact, 54 genes show a significant strain-by-age effect in our analysis (Additional file 5: Table S3).

### Egg chamber transcripts from the mitochondrial genome are significantly downregulated with age across both strains

Some sets of genes and gene pathways show consistent and concerted changes with age across various studies. Age-related changes in the expression of mitochondrial genes and genes associated with the electron transport chain have been consistently reported. This is most commonly observed as a decrease during aging [54–59]. In particular, this pattern has been observed in transcripts associated with the mitochondrial electron transport chain in the gonads of mice [60].

**Fig. 2:**
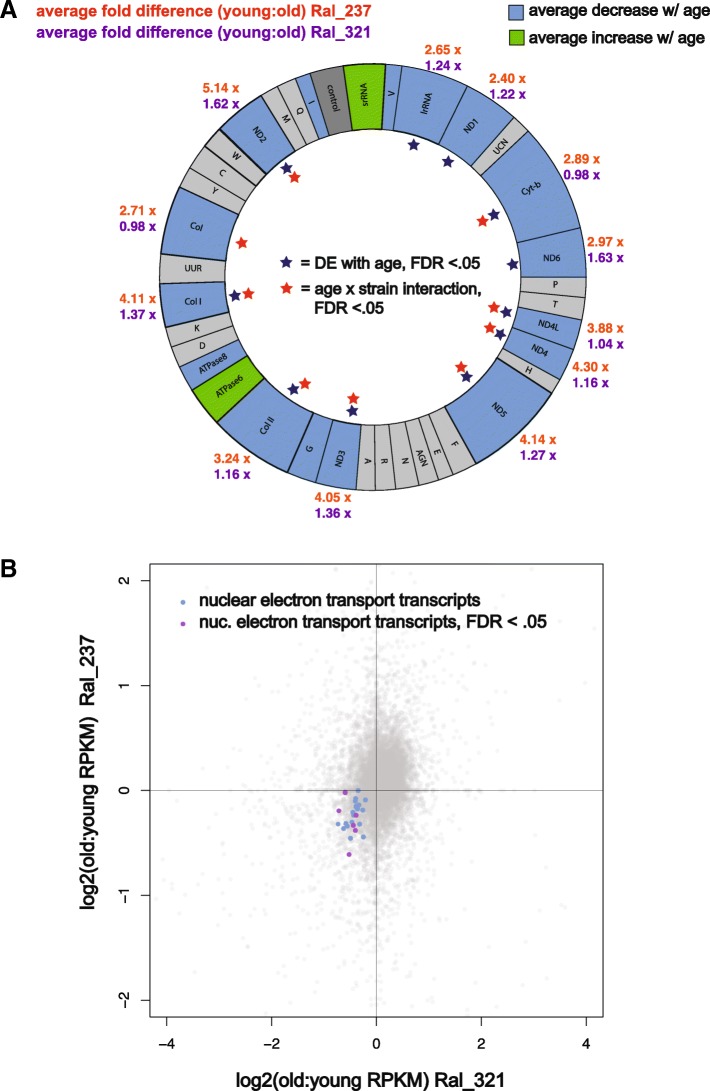
A majority of mitochondrial genome and electron transport chain transcripts decrease expression in egg chambers with age. (**a**) There is an average reduction in mitochondrial genome transcript expression in stage 14 egg chambers across strains. Some transcripts are also significant for an age by strain interaction with greater age-related fold-changes (RPKM) in Ral_321. Gray color signifies no expression or no similar change across strains. (**b**) Log2 ratios of old to young (RPKM+.5) expression between strains of electron transport chain transcripts from the nuclear genome

In stage 14 egg chambers, 11 transcripts from the mitochondrial genome significantly decreased with age in the DE analysis (Fig. 1, Fig. 2a). Nine of those transcripts also showed a significant strain-by-age effect, with greater age-related fold-changes observed in Ral_321 for seven transcripts, while two showed opposite age-related effects across the strains (Fig. 2a). In addition to transcripts from the mitochondrial genome, we also found nuclear transcripts associated with the electron transport chain significantly enriched in a gene ontology analysis (Additional file 4: Table S2). All of these nuclear electron transport chain transcripts were also downregulated with age in both strains (Fig. 2b). The downregulation of mitochondrial transcripts and those associated with the electron transport chain is in line with established mitochondrial dysfunction associated with age [40]. Our finding shows that decreased expression of mitochondrial transcripts may be a general feature of aging across all tissue types but also highlights strain-specific discrepancies in the magnitude of mitochondrial age-related effects. The reduced expression of mitochondrial transcripts in reproductive tissues may be especially significant as this could contribute to the reduced oocyte quality seen in aged flies [54–56, 59] and humans [61, 62].

### Downregulation of egg shell chorion transcripts in aged egg chambers show both shared and strain-specific effects

We found a significant gene ontology (GO) enrichment for differentially expressed transcripts associated with eggshell chorion assembly (FDR q-value < 1.44E-04, 15.4-fold enrichment). All of these DE transcripts were downregulated with age in both strains (Additional file 1: Figure S1). The downregulation of eggshell transcripts was especially striking in Ral_321, in which all but two eggshell transcripts showed a decrease with age (sign test: p-val < 1E-10; Additional file 1: Figure S1). Ral_237 also showed more eggshell transcripts downregulated with age than expected by chance (sign test *p*-value <.04) but the effect was not as strong as in Ral_321 (Additional file 1: Figure S1).

Somatic follicle cells work together to build the protective eggshell in oogenic stages 10–14. This process is dynamic, with transcript amounts changing rapidly between stages [63, 64]. Previous studies have shown that chorion transcripts from the follicle cells are expressed at very high levels in stage 14 [65]. However, due to the dynamism of late-stage oogenesis with regards to eggshell formation, we sought to confirm correct identification of stage-14 egg chambers. Tootle et al. (2011) performed a microarray analysis on 150 genes expressed in a stage-specific manner in the last 24 h of follicle development, delineated by stages 9-10a, 10b, 12, and 14. This gene expression dataset included 30 previously known eggshell genes, 19 new candidate chorion genes, and other non-eggshell or chorion genes that showed 4-fold changes in expression at late stages of follicle development. Because this gene expression dataset provides an independent temporal profile of gene expression in late-stage oogenesis, we cross-checked our egg chamber expression data against the 49 eggshell-specific transcripts. Critically, gene expression in our samples is strongly correlated with expression in stage-14 egg chambers reported in Tootle 2011 (Average across samples, Pearson’s product-moment correlation = 0.85, *p*-value < 7.80E-15) but not correlated in stages 9–10, 10b, or 12, confirming that we had captured stage 14 egg chambers in our analysis (Additional file 2: Figure S2).

The decrease in chorion transcripts with age corroborates findings of numerous other studies [43, 46, 66] and here we demonstrated that this age-effect can also vary in effect between strains. The discrepancy between the strains could be explained the fact that we used chronological age for sampling instead of physiological age. Doroszuk et al., 2012 finds that long-lived flies do not experience a typical decline of reproduction function in the later stages of life which may alternatively explain why we didn’t detect as significant of chorion effects in the strain with slightly longer median lifespan [46, 67]. Critically, this observed strain effect may also be driven by differences between the two strains in the persistence of follicle cells at this stage of egg development.

### Differentially expressed genes in egg chambers are enriched for residence in dispersed heterochromatin, but no global relaxation of heterochromatic silencing

**Fig. 3:**
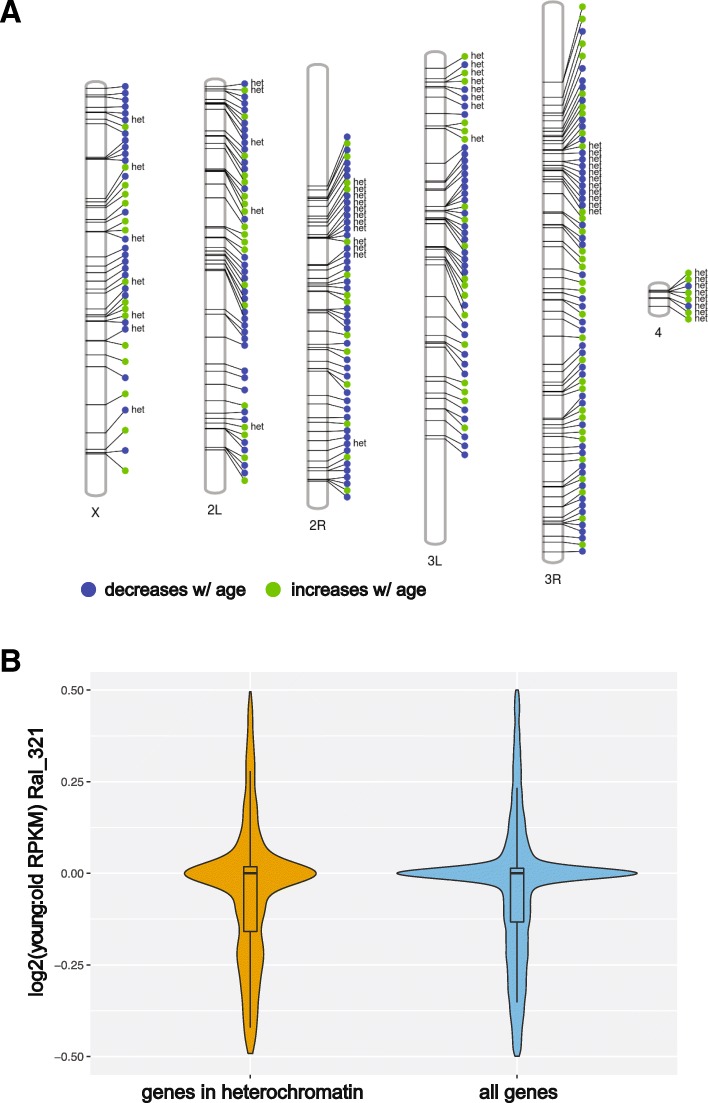
DE transcripts enriched for intercalary heterochromatin and the 4th chromosome. (**a**) Positional information of differentially expressed genic transcripts across both strains. The notation “het” indicates that the genic location intersects with heterochromatin-associated proteins, H3K9me2/me3, as reported in Kharchenko et al. 2011. DE (differentially expressed) genes located in regions of intercalary heterochromatin are marginally enriched for significance (Chi-squared with Yate’s correction, two-tailed *p*-value <.0489) but do not show a directional change of expression with age. The 4th chromosome is highly enriched for DE genes considering its limited gene composition Chi-squared with Yate’s correction, two-tailed *p*-value < .0001. (**b**) Log2(young/old RPKM) of all genes located in heterochromatin versus Log2(young/old RPKM) genome-wide expression change with age. Genes in heterochromatin show similar age-related pattern of expression change as the rest of the genome

Previous studies have implicated aberrant gene expression changes with age to changes in the heterochromatin landscape in the soma [10–15, 18]. Genome-wide expression data can be utilized as a proxy for heterochromatic changes by assessing whether genes associated with regions of heterochromatin experience age-related changes in expression. Based on previous studies, we hypothesized that genes located near heterochromatin boundaries, specifically near telomeres and centromeres, may be enriched for differential expression in aging. Kharchenko et al., 2011 described a genome-wide chromatin landscape in *Drosophila melanogaster* based on 9 prevalent combinatorial patterns of 18 histone modifications [68]. Heterochromatin domains were characterized by high levels of H3K9me2/me3. We determined the intersection of locations of our gene set with the heterochromatin regions described in that study. Of the significantly differentially expressed egg chamber transcripts across both strains in age, we found enrichment for genes in locations of intercalary heterochromatin (Fig. 3a, Table 2; Chi-squared with Yate’s correction, two-tailed *p*-value = 0.0489). We also found a striking enrichment for differentially expressed genes on the fourth or “dot” chromosome, which is primarily heterochromatic and carries only 84 genes (Chi-squared with Yate’s correction, two-tailed p-value < .0001) (Table 3). Other than the enrichment for genes on the dot chromosome, there was no obvious signature of enrichment for differentially expressed genes specifically in pericentric heterochromatin (Fig. 3a). Critically, we find that the nature of expression change with genes associated with heterochromatin is not in one direction. Differentially expressed genes associated with heterochromatin both increase and decrease during aging (Fig. 3a). This is unexpected under the standard heterochromatic aging hypothesis where heterochromatin function becomes lessened and heterochromatic genes become derepressed. Therefore, while heterochromatic regions of the genome tend to be enriched for genes that change in expression during aging, we find no release from silencing per se. To test whether there was also a subtle derepression of genes located in heterochromatin genome-wide, we compared age-related expression of all genes which overlapped with heterochromatin in the genome. We found no significant difference in the gene expression ratios between young and old egg chambers of genes located in described regions of heterochromatin compared to the rest of the genome (Fig. 3b) (*t* test for difference in mean fold difference, RAL 321 *p*-value = 0.234, RAL 237 p-value = 0.0811).

**Table 2:** Test for heterochromatin location enrichment for genes significantly DE with age

	Heterochromatin	Non-heterochromatin	Total
Significant	47	253	300
Not significant	1648	12,341	13,989
Total	1695	12,594	14,289
	***p*** **-value = 0.0489**		

Chi squared with Yate’s Correction: Chi squared = 4.494 with 1 degrees of freedom. Two-tailed test

**Table 3:** Test for enrichment of 4th chromosome genes significantly DE with age

	Dot	Other Chromosomes	Total
Significant	8	292	300
Not significant	76	13,913	13,989
Total	84	14,205	14,289
	***p*** **- value < 0.0001**		

Chi squared with Yate’s Correction: Chi squared = 19.172 with 1 degrees of freedom. Two-tailed test

We also examined whether the strain specific age-related changes for genes in intercalary heterochromatic regions were due to euchromatic TE insertions that differed between strains. It has been shown that some euchromatic TE insertions can nucleate heterochromatin formation through piRNA targeting [69, 70]. We used the DGRP strain-specific TE insertion data from TIDAL-fly [71] to compare TE insertion locations across the two strains. However, we did not identify strain-specific differences in TE insertions that correlated with aging effects that varied between the two strains.

### No global release of transposable element expression in aged egg-chambers

**Fig. 4:**
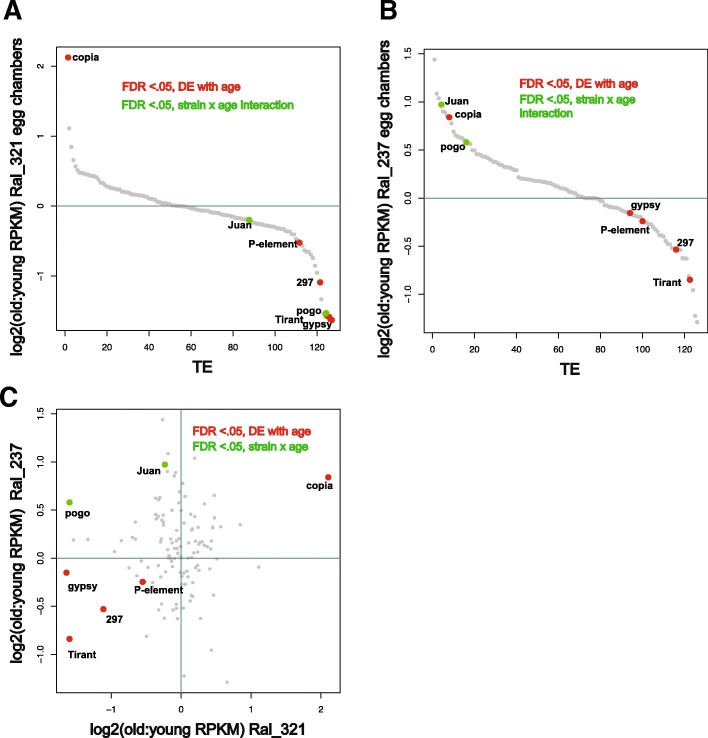
No global derepression of TEs in egg chambers from aged females. (**a**) TEs ordered by ratios of expression from old to young egg chambers in Ral_321. TEs significantly differentially expressed with age in Ral_321 tend to decrease with age. (**b**) TEs ordered by ratio of expression in Ral_237. Ral_237 shows differentially expressed TEs intercalated through a broader distribution of TE expression. (**c**) Log2 ratios of old to young RPKM+.5 of TE expression do not show a correlation with age across strains. Two TEs, *pogo* and *Juan* show significant age by strain interactions

Previous studies have shown that transposable elements become derepressed in the soma during aging, notably in brains and fat body of *Drosophila*, and in a variety of other organisms including mammals [14, 16–20]. However, a recent study on sequencing artifacts have called some of these results into question [72]. Because TEs and small RNA mechanisms of genome defense are primarily expressed in the germline, we sought to determine whether TE derepression during aging occurs in reproductive tissues in which they are primarily active. Since TE profiles can significantly differ between strains, we performed this analysis in two different strains. Using this approach, we sought to test whether results were robust to strain differences in TE profile. In contrast to other studies, we found no global TE derepression in egg chambers (paired *t* test of average expression values, young vs. old: RAL 237: *p*-value = 0.4076, RAL 321: p-value = 0.4038). While one transposable element, *copia*, increased with age across both strains, the other four TEs that showed significant differential expression with age across strains decreased in expression (Fig. 4, Table 4). Additionally, two TEs, *pogo* and *Juan*, showed a significant strain-by-age effect, exhibiting opposing directions of expression with age across the strains (Fig. 4c). This may be attributed to differences between strains in the abundance and location of *pogo* and *Juan* insertions. Figure 4c also illustrates that the TEs that are significant in Ral 321 are dispersed throughout the wider distribution of TE expression for Ral 237. There is also no significant correlation between the ratio of TE expression between young and old egg chambers across strains (Fig. 4c, *p* = 0.4851). This lack of correlation indicates that variation in the TE profile between strains can have a strong influence on the effect of aging.

**Table 4:** Differential expression results for TEs

*TE*	*EGG CHAMBER*				
	fold difference			FDR adj. *p*-value	
	*Ral_321*	*Ral_237*	*drxn w/ age*	*age*	*strain x age*
*Tirant*	3.02	1.80	down	**0.007**	0.940
*297*	2.13	1.45	down	**0.010**	0.869
*Gypsy*	3.10	1.11	down	**0.011**	0.125
*Copia*	4.36	1.79	up	**0.034**	0.849
*P-element*	1.44	1.19	down	**0.041**	0.939
*Pogo*	2.99	1.49	down, up	0.125	**0.027**
*Juan*	1.16	1.96	down, up	0.597	**0.039**

TEs that show significant differential expression with age in egg chambers. Two TEs show a strain-by-age interaction. Fold change refers to fold change differences in RPKM levels, as indicated by direction of change with age (drxn w/age). Bold indicates *p*-value < 0.05

### piRNA pathway transcripts are increased in aging egg chambers

**Fig. 5:**
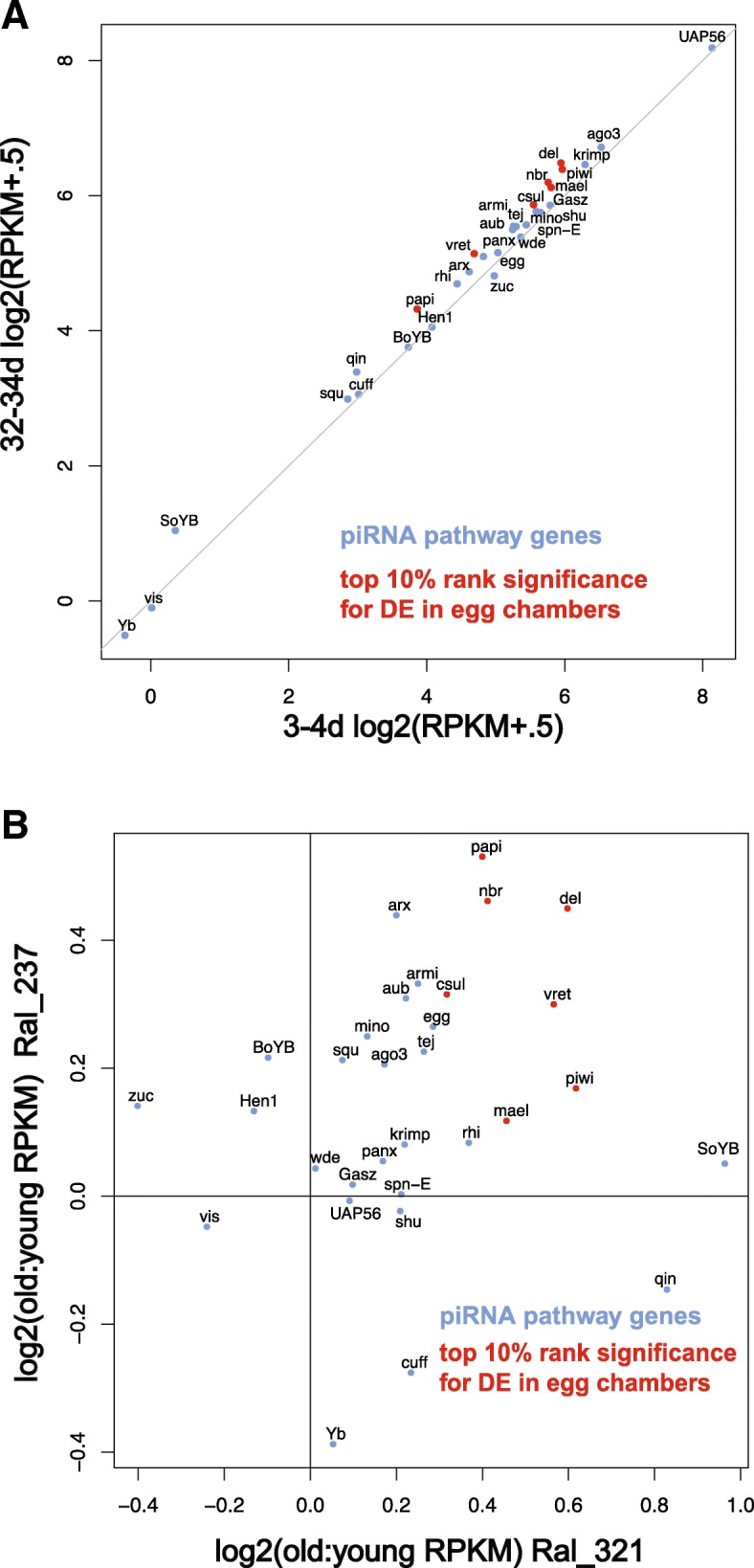
piRNA transcripts increase with age in egg chambers. **a**) Expression (RPKM+.5) of piRNA pathway transcripts between egg chambers of young and old females. Red dots indicate transcripts that were in the top 10% of significant FDR-adjusted *p*-values. **b**) Log2 ratios of old to young piRNA pathway transcript expression (RPKM+.5) in egg chambers across strains. Both strains show a that a majority of piRNA transcripts increase with age. Relative to similarly expressed genes, the piRNA machinery shows a significantly higher level of expression in older egg chambers (permutation test, *p* = 0.0131)

TE control by piRNA in the germline has been shown to be sensitive to aging. Syndromes of hybrid dysgenesis reveal that the sterilizing effects of an activated TE can become ameliorated as flies age [73–76]. This has been attributed to an increased capacity for TE fragments residing in heterochromatin to contribute to the piRNA pool in older flies [77]. Moreover, this effect of aging can be transmitted across generations since maternally transmitted piRNA pools establish piRNA biogenesis in offspring. Since some TEs did show significant decreases with age, we tested whether genes in the piRNA pathway, which regulate TE expression in the *Drosophila* germline, showed any age-related expression changes in egg chambers. Strikingly, 27 out of 31 piRNA pathway genes show an average expression increase with age combined across the two strains (Fig. 5a). As expected, the somatic Yb transcript is expressed at a very low level in stage 14 egg chambers. To test significance, we performed a permutation test where each piRNA gene was matched for the 20 most similarly expressed genes (10 ranked higher, 10 ranked lower). With these matched genes, we determined the proportion of random samples of gene sets where the average fold increase was equal to or greater than that observed for the piRNA machinery in both strains (Fig. 5b), revealing that an increase in expression of the piRNA machinery is significant (*p* = 0.013). piRNA genes are also enriched in the top 10% of differentially expressed transcripts ranked significance (Chi squared with Yate’s correction, *p*-value = .044). Variation in the expression of the piRNA machinery has been previously linked to variation in TE expression [78–80].

## Discussion

With delays in childbearing on the rise, the study of reproductive decline grows increasingly relevant [1]. Fruit flies are an excellent model organism to study because they experience a clear reproductive decline, existing age-related literature in flies is vast, and *Drosophila* share several mechanisms and pathways in ovulation and gametogenesis with mammals [5].

Genome-wide RNA-seq studies have shown that different tissues vary in age-related signatures, highlighting the importance of analyzing each tissue individually in each species [42, 60]. Reproductive tissues are unique in that they are a mix of interacting somatic and germline tissue. While the germline is widely recognized as being more resistant to aging than somatic cells, some age-related changes are known to occur. Critically, the relative contribution of factors intrinsic versus extrinsic to the germ line in reproductive decline remains poorly understood.

Here, we report a set of genes that show similar changes across genetic backgrounds in aged egg-chambers. We additionally highlight the role genetic background plays in age-related effects. For example, while the decline we show in chorion-related transcripts with age parallels other studies, we demonstrate that the severity of this age-effect depends on genetic background. This may be explained by either differences in the regulation of chorion genes or differences in the persistence of follicle cells in egg chambers of the two strains.

We also show that aging in late-stage egg chambers can mirror that of other tissues, with downregulation of transcripts from the mitochondria and nuclear transcripts associated with mitochondrial activity. Oocytes have significantly more mitochondria than any other cell, highlighting the significant energy demands of gametogenesis [81]. The dysfunction of oocyte mitochondria has been proposed as a possible mechanism involved in reduced competence of oocytes in older human infertility patients [61]. One of the most well documented age-effects thought to reduce female fertility is aneuploidy. Reduced mitochondrial activity may contribute to this decline, as improper chromosome segregation has been induced in oocytes deficient in mitochondrial enzymes that metabolize pyruvate [62]. Our results support the idea that mitochondrial age effects could contribute to reproductive decline. Changes in mitochondrial ultrastructure have been observed in aging flies [82]. Because mitochondria are maternally transmitted, the possible deposition of abnormal mitochondria with advanced age has been hypothesized to negatively contribute to offspring health.

Epigenetic changes have also been implicated as playing an important role in the aging process in cells of the soma across model organisms. Specifically, genome-wide heterochromatin redistribution during aging has been linked to the derepression of transposable elements and an overall loss of gene regulation. Whether or not epigenetic factors are perturbed in reproductive and germline tissues is of particular interest because some epigenetic factors are known to transmit across generations [37, 39, 77]. While several studies have reported aberrant gene expression in aging on a genome-wide scale [10, 14, 15], we report no overall loss of gene regulation in aged egg chambers, consistent with another *Drosophila* study using whole bodies [43]. Previously, it was shown that reporter genes residing in heterochromatin regions of the fly experienced loss of silencing with age [15]. In line with these findings, we find that genes that show significant age-related expression differences are enriched for regions of heterochromatin, especially on the fourth chromosome, providing evidence for age-related epigenetic changes occurring in late-stage egg chambers of *Drosophila* oogenesis. However, we do not find evidence that the landscape of heterochromatic silencing is relaxed in older egg chambers. Instead, during aging, genes residing in heterochromatin show both increases and decreases in expression. Interestingly, the mitochondria has been shown to have an impact on the nuclear epigenome [83]. Thus, some of these changes may be influenced by changes in the expression of mitochondrial transcripts that we also observed.

Differences between somatic and reproductive tissues in how gene expression changes during aging may also extend to transposable elements. A decline in repressive heterochromatin with age has been associated with TEs becoming active and mobile in aging somatic cells [16, 20]. Because increased transposition promotes DNA damage and increased mutagenesis, age-related transposable element derepression has been proposed to be an important component of genomic instability and a contributor to the prevalence of disease that accompanies advanced age. Here, we find no evidence that TEs are derepressed as a general feature of aging in egg chambers. In contrast, we find that the handful of TEs that are differentially expressed with age tend to *decrease* in expression with age, in conflict with current TE aging theories, but in line with the idea of improved adaptive piRNA-mediated immunity with age [75, 77].

Studies have shown that the expression of the piRNA machinery may be linked to variation in TE expression [78–80]. Overall, our results indicate that an important determinant of variation in the expression of the piRNA machinery may be age itself. In contrast to what might be expected in the aging soma, increased expression of the piRNA machinery in aging reproductive tissues suggests that aging may lead to increased investment in germline genome stability. Interestingly, a decrease in expression of the piRNA machinery during aging was observed in worker termites [22]. The increase in expression in piRNA pathway genes reported here identifies a difference compared to non-reproductive tissues.

Increased expression of the piRNA machinery may be a general feature of the aging ovary in *Drosophila* that occurs simply over time, but is independent of the direct effects of aging itself. Or, instead, it may be the result of a regulatory response to compensate for negative effects that accumulate during the aging process. A recent study has shown that aging allows the piRNA machinery to compensate for depletion of Aubergine protein from dividing germline cells in the germarium [84]. One effect of aging that might influence the expression of the piRNA machinery is the age-dependent decrease that we found for mitochondrial transcripts. Critical aspects of piRNA biogenesis are known to occur at the mitochondrial surface [85–88]. Thus, if mitochondrial function is impaired, there may be a programmed response to increase expression of the piRNA machinery in the ovaries of older flies. Though the mechanism is currently unknown, increased investment in germline stability in older *Drosophila* appears to contrast with what is observed in humans, where female aging leads to an increase in chromosomal non-disjunction and male aging leads to an increased mutation rate.

## Conclusions

In summary, here we show that there is evidence for diverse age-related change within the reproductive tissues and germline of *Drosophila melanogaster*. However, these tissues are more robust to age-related change in gene expression than the soma, as we find no global TE derepression or global relaxation of heterochromatic silencing with age. Instead, despite the fact that mitochondrial function appears to decrease, the increasing expression of the piRNA machinery indicates there might be increasing investment in maintaining genome stability during the aging process. This study supports the conclusion the germline is generally robust to age-related epigenetic changes.

## Methods

### Fly stocks and stage 14 egg chamber tissue collection

*D. melanogaster* DGRP [49] lines RAL-237 and RAL-321 were utilized for this study and maintained at 22 degrees Celsius and 12- h light cycles. Flies were maintained in bottles at controlled larval density (~ 100 per bottle) for two generations before tissue collections. Approximately thirty zero-to-one day old third generation females were transferred to individual vials for aging treatment and supplemented with two males ranging from 3 to 7 days old approximately every seven days to encourage egg production. Flies were moved to fresh vials weekly. Stage 14 egg chambers were identified visually through their elongated dorsal filaments. In *Drosophila,* mature oocytes are activated in preparation for embryogenesis, when they pass through the female oviduct. To ensure activated eggs weren’t selected, oocytes were directly extracted from the ovary. Unactivated stage 14 oocytes have a limp and deflated appearance, while activated eggs are firm and more opaque [89]. For each pooled replicate, stage 14 egg chambers were dissected from ovaries of five 3–4 and five 32–34 day old females in PBS buffer. Using a thinly bristled paintbrush, 2–5 egg chambers from each female were added to single caps of .2 mL tubes, stabbed with RNAse free needles in 30uL TRIzol, and flash frozen in liquid nitrogen. Overall, approximately 20 pooled egg chambers from 5 different females were collected for each of the biological replicates. Correct staging was also confirmed by RNA-seq analysis (Additional file 2: Figure S2) by comparing to the results of a previous study [63].

### RNA extraction and mRNA sequencing

For RNA extraction, egg chambers from 5 females (~ 20 egg chambers total per biological replicate) were pooled. Accounting for the TRIzol already in the samples from the collection stage, we added up to a total volume of 300uL TRIzol for RNA extractions. To improve recovery in the separation phase, we used 5PRIME Phase Lock Gel Heavy tubes. RNA was resuspended in 25uL of water. Library preps were performed using the NEBNext Ultra Kit according to the manufacturer’s instructions (New England Biolabs). NEBNext Ultra libraries were pooled in groups of 8–10 per lane, and run with single-end 100 bp reads on a HiSeq 2500.

### Analysis of mRNA sequencing data

Read mapping and RNA-seq analysis was performed using CLC Genomics Workbench 8 using release 6 of the *Drosophila melanogaster* reference genome. For expression values, RPKM estimates generated by the RNA-seq tool in CLC Genomics Workbench were used. FDR-adjusted *p*-values for significant differential expression using count values were calculated with the CLC implementation of the DESeq2 package in Bioconductor [90]. Using DESeq2, we determined significance for treatment effects (age), strain effects and strain-by-treatment effects. To estimate TE family expression, the annotated *D. melanogaster* TE library (ftp://ftp.flybase.org/releases/FB2018_04/precomputed_files/transposons/transposon_sequence_set.embl.txt.gz) was appended to a genome sequence masked for individual TE sequences. GO analysis was performed with GOrilla [53] using *D. melanogaster* orthologs genes sorted by FDR p-value for the test of treatment effect.

## Additional files

Additional file 1: **Figure S1.** Transcripts associated with the eggshell are downregulated with age in both strains but show stronger age effects in Ral_321. Log2 ratios of expression (RRKM + .5) of transcripts associated with the eggshell between young and old egg chambers across strains. (PDF 7045 kb)

Additional file 2: **Figure S2.** Verification of stage 14 transcript expression. Transcripts that show stage-specific expression in final stages of oogenesis as defined by Tootle et al. 2011. Transcript expression from stage 14 egg chambers is strongly correlated with stage 14 oogenic-specific transcript expression but not with the other stages in Tootle et al., 2011. (PDF 969 kb)

Additional file 3: **Table S1.** RPKM values for individual samples, averages, and direction of expression change with age. *P*-values for differential expression in age, controlled for strain also provided. (XLSX 126 kb)

Additional file 4: **Table S2.** Gene ontology (GO) analysis for biological process, component, and function, by rank significance. (XLSX 2404 kb)

Additional file 5: **Table S3.** Gene RPKM values for individual samples with *p*-values for strain-by-age effects. (XLSX 2403 kb)

## Abbreviations

DE: differentially expressed
DGRP: *Drosophila* Genetic Reference Panel
GO: gene ontology
RPKM: reads per kilobase mapped
TE: transposable element
