# Supplementary figures and images for: Aging in the Drosophila ovary: contrasting changes in the expression of the piRNA machinery and mitochondria but no global release of transposable elements
Source: BMC Genomics. 2019 Apr 23;20:305. doi: 10.1186/s12864-019-5668-3 (PMC6480902; doi:10.1186/s12864-019-5668-3)

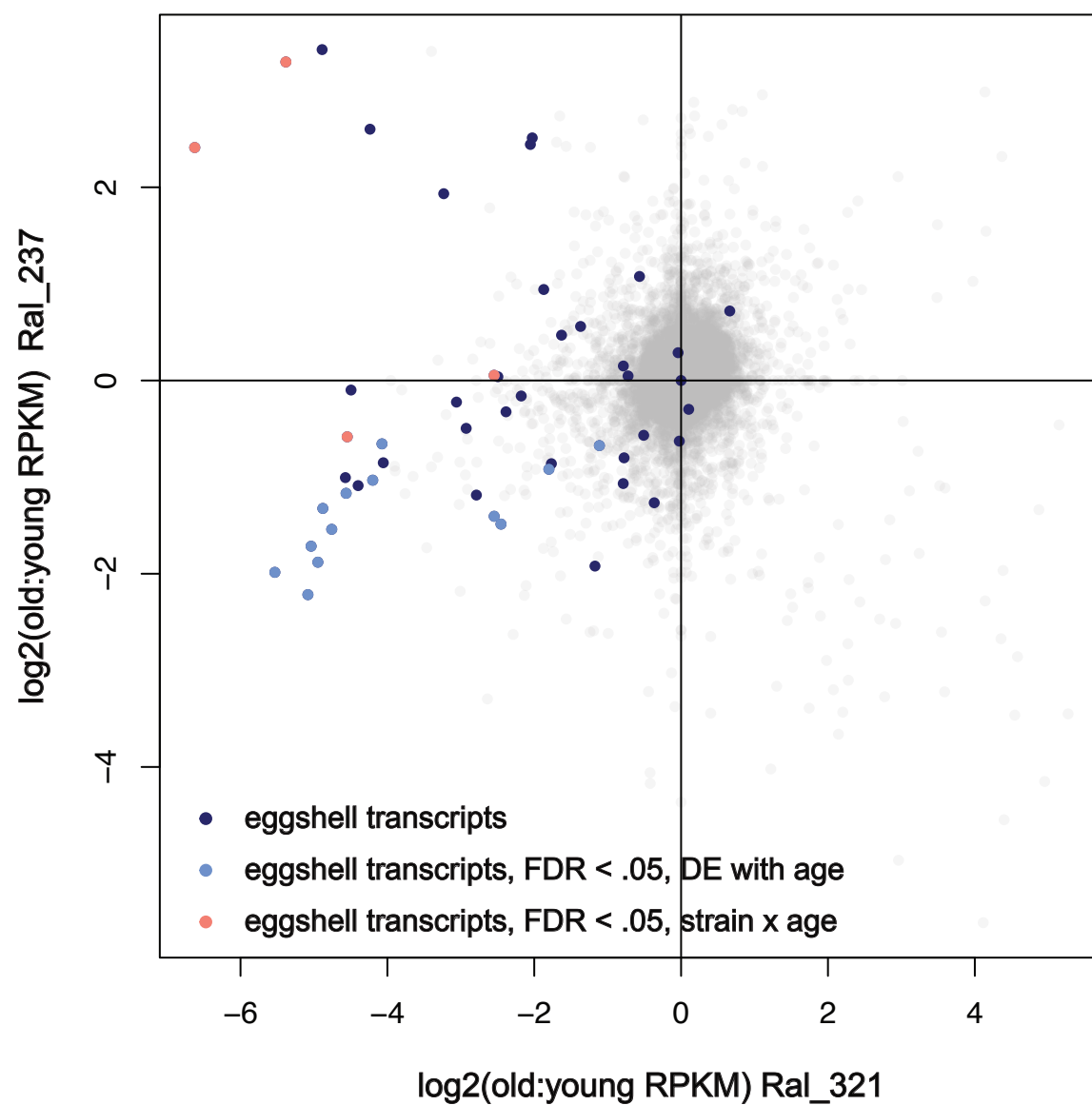

Supplement: Supplementary file 1 — Figure S1. Transcripts associated with the eggshell are downregulated with age in both strains but show stronger age effects in Ral_321. Log2 ratios of expression (RRKM + .5) of transcripts associated with the eggshell between young and old egg chambers across strains. (PDF 7045 kb) [file 12864_2019_5668_MOESM1_ESM.pdf]

Tootle et al. relative expression (microarray)

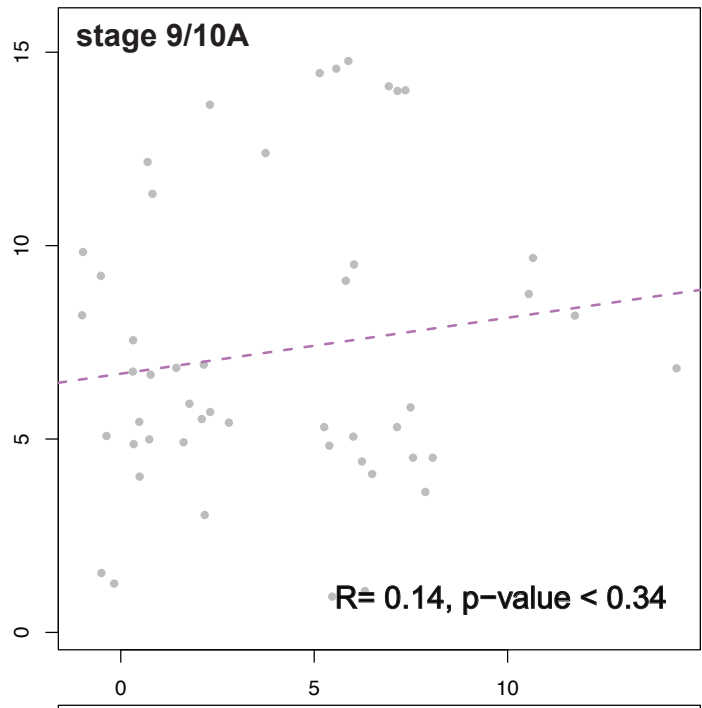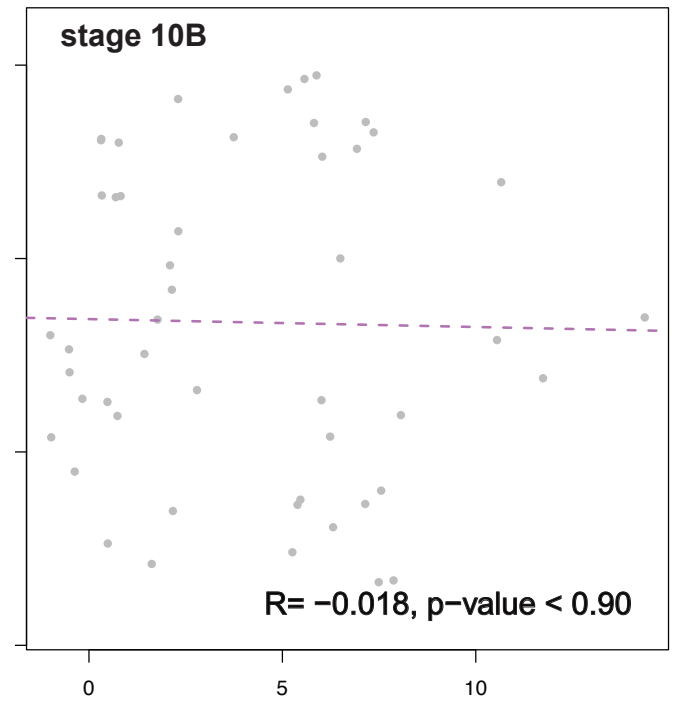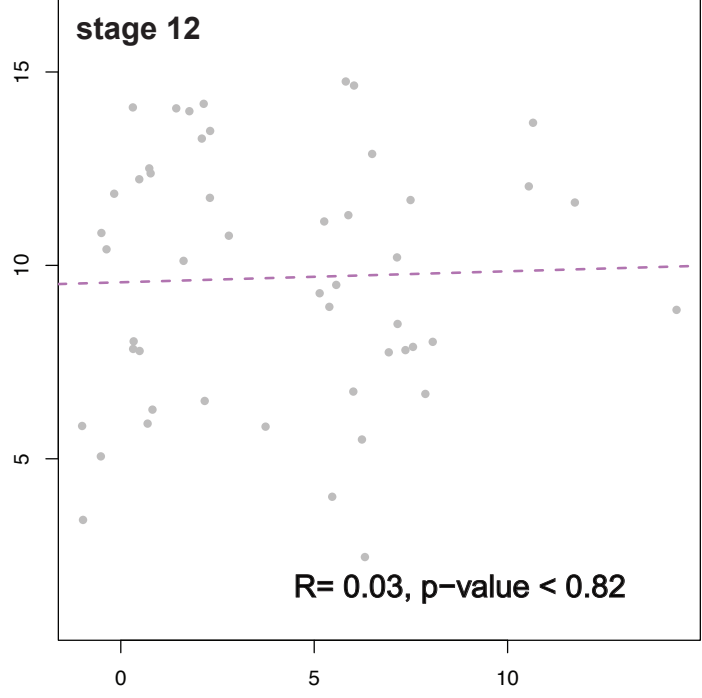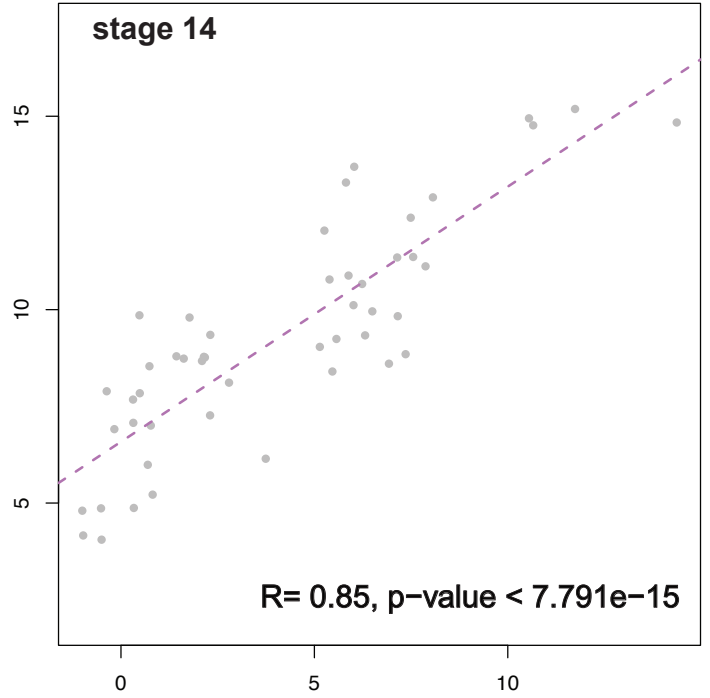

3-4d Ral\_237 & Ral\_321 egg chambers (avg RPKM), stage 14

Supplement: Supplementary file 2 — Figure S2. Verification of stage 14 transcript expression. Transcripts that show stage-specific expression in final stages of oogenesis as defined by Tootle et al. 2011. Transcript expression from stage 14 egg chambers is strongly correlated with stage 14 oogenic-specific transcript expression but not with the other stages in Tootle et al., 2011. (PDF 969 kb) [file 12864_2019_5668_MOESM2_ESM.pdf]
